# Editorial: Stem cells and the immune system

**DOI:** 10.3389/fcell.2023.1310268

**Published:** 2023-10-19

**Authors:** John M. Perry, Meng Zhao

**Affiliations:** ^1^ Children’s Mercy Kansas City, Kansas City, MO, United States; ^2^ Department of Pediatrics, University of Kansas Medical Center, Kansas City, KS, United States; ^3^ Department of Pediatrics, University of Missouri Kansas City School of Medicine, Kansas City, MO, United States; ^4^ Key Laboratory of Stem Cells and Tissue Engineering (Ministry of Education), Zhongshan School of Medicine, Sun Yat-Sen University, Guangzhou, China

**Keywords:** stem cells, cancer stem cell (CSC), immunity, immune system, immune privilege, immunotherapy, microenviroment, niche

A dynamic interplay between *stem cells and the immune system* can result in cancer development. Recent research has revealed that cancers can be initiated, sustained, and rekindled by a relatively rare stem or stem-like subpopulation of cells. Fortunately, a highly potent immunosurveillance system eliminates these tumorigenic cells as they emerge. However, recent research suggests that cancer stem cells are particularly potent in overcoming immune barriers and are thus especially resistant to natural anti-cancer immunity and the clinical interventions of immunotherapy. Thus, stem cell dialoge with the immune system is a particularly critical topic regarding the origin and successful treatment of cancer. Similarly, these concepts are particularly important since recurrence or relapse continues to represent the main barrier for effective treatment, including otherwise highly promising immunotherapeutic strategies. Regrettably the underlying mechanisms for this resistance continue to be only poorly understood, although recent studies have begun to enlighten this emergent field.

This Research Topic features the emerging field of stem cells in their immunological context. Stem cells are not merely discrete, autonomous cellular entities but are influenced and governed by their microenvironment. Here, the immune system’s role in normal and tumorigenic microenvironments is explored, particularly with regards to stem cells and the genetic signaling pathways governing crosstalk between stem and immune cells. Furthermore, healthy stem cells can adopt an immune privileged state which may be hijacked by cancer stem cells. Notably, understanding healthy stem cells and their crosstalk with immune cells will enlighten how tumorigenesis corrupts normal stem-immune cell processes.

In this Research Topic, Wong and Sugimura review immune-epigenetic crosstalk in hematological cancers. Since the immune system represents a major developmental product of hematopoiesis, and hematological malignancies are often driven by subverted epigenetic and immunological mechanisms, these cancers represent an ideal system for studying immune-stem cell interactions. In particular, chronic inflammation caused by inflammatory cytokines secreted by tumor cells and healthy, proximal cells in the bone marrow microenvironment activates critical transcription factors, which can result in the origination of cancer stem cells and resultant disease progression. As discussed by Wong and Sugimura, these critical factors include the Smad family, STAT3, and NF-κB. Understanding the interaction between immune cells and malignant cells, and the resulting epigenetic modifications, such as DNA methylation and histone modification, is expected to broaden the horizon for new, targeted therapies. Combining epigenetic therapy with immune checkpoint inhibitors is also expected to enhance treatment efficacy, particularly in relapsed patients. However, the complex nature of the bone marrow microenvironment and the heterogeneity of cancer cells require a personalized medicine approach, particularly the development of biomarkers identifying the most suitable therapies for different patient subtypes and even different stages of progression in the same patient. Overall, this review provides a timely, emergent synthesis of two critical topics in oncology research.

Also regarding crosstalk between stem and immune cells, Fan et al. provide a review of the role of macrophages and bone marrow mesenchymal stem cells (BMSCs) in bone healing—a fascinating topic broadly conceptualized as osteoimmunity. The role of macrophages and BMSCs in bone healing and their interaction in regulating the balance between inflammation and regeneration is highlighted in this review. In particular, when this interaction is disrupted, it can lead to a failure of bone regeneration, in part by disrupting BMSCs. This review dissects the mechanism and significance of macrophage-BMSC interaction and explores new therapeutic ideas for targeting this interaction to regulate the inflammatory response in bone healing. Overall, the role of macrophages and BMSCs in bone healing and the need for a deeper understanding of their interaction is emphasized. Promisingly, the authors highlight how targeting macrophage-BMSC crosstalk, particularly with regard to regulating the relevant inflammatory responses in the injury *versus* healing processes, could provide new directions for promoting bone restoration.

Soliman and Abdellatif review mesenchymal stem cells originating from a different tissue—those harvested from the umbilical cord. As the umbilical cord is typically discarded, cells derived from this tissue are readily available without additional medical interventions or side-effects. Notably, UC-MSCs have extensive immunomodulatory properties, leading to the exploration of their use for alleviating effects from acute infection, including the recent pandemic causing COVID-19. In particular, cytokine release syndrome or cytokine storm is associated with dramatically increased risk for morbidity and mortality from COVID-19. This review discusses how UC-MSCs may suppress and even control adaptive and innate immune responses by attenuating cytokine release syndrome resulting from SARS-CoV-2 infection.

Finally, Chavez et al. provide an original research article on the essential role of PU.1 in restraining myelopoiesis during chronic inflammatory stress. Chronic inflammation, sustained by the immune system, is a shared property of aging and numerous disease states, including the development of hematological malignancies. Hematopoietic stem cells (HSCs) are the ultimate source for myeloid cells that can damage tissues by inducing and sustaining inflammatory states. HSCs with biased differentiation for such inflammatory cells thus serve as the source of this condition. Furthermore, HSCs accumulate mutations and sometimes stable epigenetic states over an organism’s lifespan. Thus, mutant or even normal transcription factors can access aberrant transcriptional sites and potentially wreak havoc on genetic signaling pathways, ultimately driving leukemogenesis. PU.1 is well-known a “master regulator” transcription factor, which is induced in HSCs by pro-inflammatory signals. The authors found that PU.1 restrains inflammatory myelopoiesis. This result may be unexpected since PU.1 is known to be a key driver of myeloid differentiation. However, Chavez et al. show that PU.1 adapts HSCs and their early downstream progenitor responses to inflammatory stimuli. Overall, PU.1 is revealed to regulator “emergency” myelopoiesis with implications for chronic inflammatory diseases as well as leukemia development.

Overall, this Research Topic highlights how cancer develops only in the context of immune breakdown. Understanding how stem cells normally interact with the immune system and how cancer cells escape immune recognition will be critical for future advances in cancer prevention and developing enduring cures. Here, the current state of knowledge for this emerging field of stem cells in the context of the immune system is explored, illuminating novel targets for improving the current armamentarium of cancer immunotherapy.

